# Clinical efficacy analysis of percutaneous “tripod” combined with radiofrequency ablation and bone cement filling in the treatment of periacetabular metastases

**DOI:** 10.1186/s13018-023-04255-w

**Published:** 2023-10-11

**Authors:** Yun Lan, Ruoyu Li, Linheng Jiang, Nannan Zhou, Mincon He, Bin Fang, Chunzhi Yi

**Affiliations:** 1 Department of Orthopedic Oncology, The First Affiliated Hospital of Guangzhou University of Chinese Medicine, Guangzhou, 510000 Guangdong China; 2 The First Clinical Medical College of Guangzhou University of Chinese Medicine, Guangzhou, 510000 Guangdong China; 3 Guangdong Academy of Traditional Chinese Medicine Orthopedics and Traumatology, Guangzhou, 510000 Guangdong China

**Keywords:** Acetabulum, Metastases, Tripod, Radiofrequency ablation, Bone cement

## Abstract

**Background:**

To investigate the clinical efficacy of a percutaneous “tripod” combined with radiofrequency ablation and bone cement filling surgery in treating acetabular bone metastases.

**Methods:**

We retrospectively analyzed 11 patients who underwent percutaneous “tripod” combined with radiofrequency ablation and bone cement filling for acetabular bone metastases at a tertiary care hospital from February 2021 to December 2022.

**Results:**

11 cases with 13 hips underwent this procedure, including two female patients who underwent both sides, and the rest were unilateral. All cases were followed up for 3–24 months, with a mean of 12 months and a median follow-up time of 11 months. Two of the 11 patients died by the final follow-up, and nine survived. One died 7 months after surgery, and one died 8 months after surgery; the survival of the deceased patients was 7.5 months (range: 7–8 months), with a median survival time of 7.5 months. All 11 patients completed the surgery successfully, and the average unilateral operation time was 167.4 min (148–193). The amelioration of postoperative pain, concomitant with improved quality of life, was observed significantly, ultimately resulting in a prolonged and sustained effect.

**Conclusions:**

The combination of percutaneous “tripod”, radiofrequency ablation, and bone cement filling can effectively relieve pain without delaying the patient's systemic anti-tumor therapy and is a minimally invasive, safe, and effective procedure for the treatment of periacetabular metastases.

**Supplementary Information:**

The online version contains supplementary material available at 10.1186/s13018-023-04255-w.

## Background

In recent years, the incidence of bone metastases has gradually increased with the improvement of the efficacy of patients with malignant tumors, and the pelvis is a common bone metastasis. When bone metastases around the acetabulum, it will cause severe pain, limited hip movement, inability to stand or walk, and even long-term bed rest, accelerating the deterioration of the patient's condition and seriously affecting the patient’s quality of life.

For periacetabular metastases, surgery is one of the options. Harrington [1] reported in 1981 the treatment of periacetabular bone metastases, dividing periacetabular metastases into four types and designing remedies for each class. It has created a precedent and standard for treating periacetabular bone metastases, and most subsequent scholars have adopted this classification and treatment method and achieved specific clinical effects. Among them, for Harrington type III patients, the reconstruction method of using Steinmann pins combined with bone cement to strengthen the acetabulum and transmit stress to the sacroiliac joint through Steinmann pins (Harrington procedure) has been recognized and used by many scholars. However, the area is anatomically complex, excision and reconstruction are complicated, and postoperative infection, loose or dislocated prosthesis, and high fractures [2–5]. At the same time, patients have to suspend antitumor therapy perioperatively due to open surgery, which may delay the systemic, comprehensive treatment of malignant tumors, leading to tumor progression. In addition, some patients with acetabular metastases are older and have poor general conditions, making complex open surgery difficult. Therefore, the selection and implementation of surgical protocols for periacetabular metastases are complicated, and there is no unified standard.

The relevant literature search and the analysis of acetabular mechanics show that the acetabulum is the leader of the mechanical transmission and support structure. The acetabulum is composed of the fusion of iliacia, ischia, and pubis, and its anterior and posterior columns form similar inverted “Y” shaped support. The pain of patients with periacetabular metastases is likely caused by structural instability and pathological fractures. Therefore, enhancing the stability and mechanical transmission of the anteroposterior column of the acetabulum can better relieve the patient's pain and mobility function. In 2020, Rui Yang et al. [6] reported percutaneous tripod surgery for treating periacetabular metastases with satisfactory results. Based on this surgery, we used a percutaneous “tripod” combined with radiofrequency ablation bone cement filling to treat periacetabular metastases, and the efficacy of this procedure is reported here.

## Patients and methods

The clinical study was conducted at the Bone Oncology Center of a tertiary hospital. 11 patients were enrolled in the present study according to the inclusion and exclusion criteria. The inclusion criteria are as follows: (1) patients with pelvic metastases diagnosed clinically, imaging, and pathologically; (2) Metastatic lesions around the acetabular: Harrington type III; (3) The expected survival period is more significant than 1 month. The exclusion criteria were as follows: (1) ipsilateral femoral head lesions, extensive bone destruction at the screw anchoring position; (2) those with other serious diseases or systemic evaluation who cannot tolerate surgery; (3) pain caused by direct compression of soft tissue masses or tumor invasion of nerves; (4) osteogenic lesions without fractures; (5) the estimated survival is less than 1 month. All patients had pelvic X-rays and CT scans completed before surgery. F

## Surgical techniques and follow-up protocol

### Preoperative preparation

Pelvic X-rays and CTs are required before surgery. Preoperative pelvic CT 3D reconstruction can better understand the patient’s periacetabular anatomy and bone destruction and is conducive to preoperative planning to determine the angle and position of screw insertion. Preoperative bowel preparation should be performed to improve the fluoroscopy of the pelvis and associated anatomical landmarks during surgery. All operations were performed by the same team, which consisted of two orthopaedic trauma surgeons with at least 10 years of experience. All surgery was conducted under spinal or general anesthesia. Routine prophylactic antibiotics were used 30 min before surgery.

### Surgical techniques

The patient is placed in a horizontal supine position with both upper limbs abducted to 90°. It is recommended to use a urinary catheter to reduce the filling of the bladder, thereby reducing damage to the bladder. Skin disinfection should cover the lower edge of the ipsilateral costal arch, the lower abdomen and perineum, the ipsilateral buttocks, and the lower limbs to the top of the ankle joint. The C-arm host is located on the opposite side of the surgical site.

#### Place the tripod

Under the guidance of the C-arm machine, the puncture trocar needle is inserted percutaneously at the corresponding starting point, and after the trocar penetrates the bone cortex, the angle and position are determined under fluoroscopy, the needle core is withdrawn, and the hollow screw guide needle is inserted through the trocar channel. After several C-arm machine views to ensure correct pin positioning and needle insertion trajectory, the open screw is inserted. The specific operation is as follows:

##### Anterior column screw

**Fig. 1:**
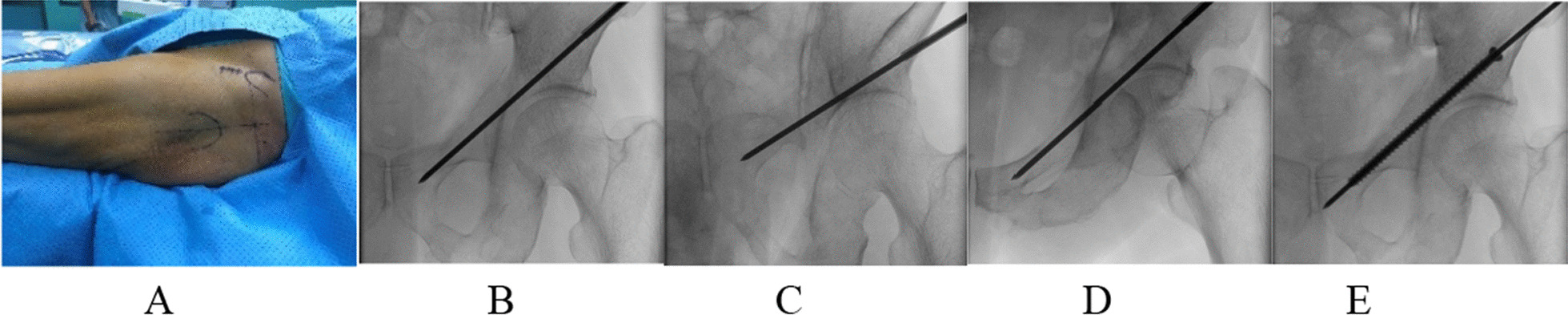
Insert the anterior column screw (**A** take the upper extension of the apex of the greater trochanteric of the femur and the horizontal intersection of the anterior superior iliac spine at the needle entry point and insert the needle in the direction of the pubic symphysis; **B**–**D** pelvic orthography, exit radiograph, and entrance position determine that the guide needle does not penetrate the cortex; **E** Insert the screw along the guide pin)

Place the screw in an antegrade manner, starting from the intersection of the upward extension line of the apex of the greater trochanter and the horizontal line of the anterior superior iliac spine, and insert it toward the pubic symphysis. Use a biopsy needle to puncture percutaneously. The operator touches the upper edge of the pubic symphysis to determine the direction of entry of the puncture needle. From the lower back to the upper front, the needle entry point and angle are determined under C-arm fluoroscopy. Then, pull out the needle core of the puncture needle, put the guide needle along the biopsy needle sleeve, and confirm that the guide needle is located in the pubic bone and does not enter the hip joint through the favorable position of the pelvis, the exit position, and the entrance position (Fig. 1). Note: 1. The needle entry point needs to be adjusted according to the patient’s obesity degree; the more obese the patient, the higher the needle entry point; 2. The needle needs to be inserted in the direction from the lower back to the upper front, towards the upper edge of the pubic symphysis, generally at an angle of 30° with the horizontal plane; 3. For patients with complete cortical bone, the guide pin can enter along the cortical bone, which is easier to complete. For patients with damaged bone cortex, the guide pin is easy to pass out during the operation and needs to be adjusted; 4. At least two X-ray films at different positions are required to confirm that the guide pin is in the bone.

##### Posterior column screw

**Fig. 2:**
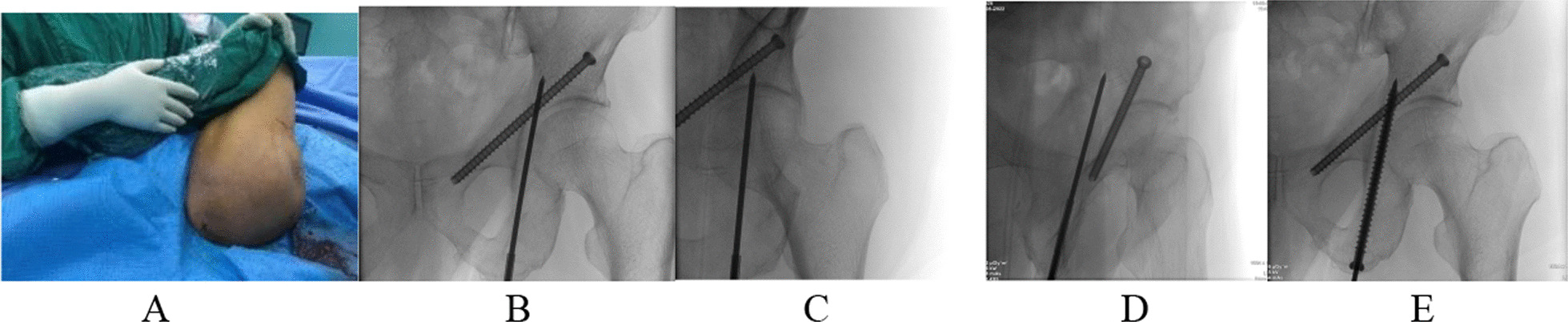
Posterior column screw (**A** The extreme flexion position of the affected hip joint, with the top of the ischial tubercle as the needle entry point, the needle is inserted in the direction of the medial edge of the sizeable ischial notch; **B**–**D** pelvic alignment, oblique view, and ilium diagonal position determine that the guide needle does not penetrate the cortex; **E** Insert the screw along the guide pin)

The hip on the affected side is taken in an extremely flexion position, exposing the ischial tubercle, touching the top of the ischial tubercle as the needle entry point, and implanting a guide needle in the direction of the medial edge of the sizeable ischial notch. The guide needle is located within the ischia by pelvic alignment, oblique obturator position, and ilium oblique position. Note: 1. Need an assistant to help flex the hip knee, expose the ischial tubercle, the needle entry point as far as possible along the ischial apex, easy to outward, 2. The needle angle is 10° valgus from the midline, 3. When entering the needle, you need to lower the guide needle to avoid the guide needle from penetrating from the back wall, our experience is to flex the hip as much as possible to raise the ischial tubercle, and the hand is close to the bed surface when entering (Fig. 2).

##### Transcolumn screws

**Fig. 3:**
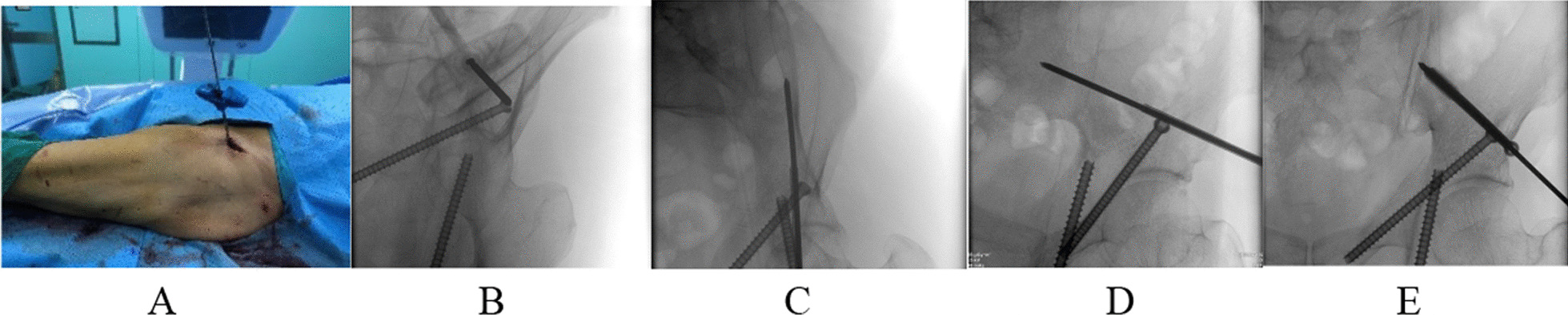
Placement of trans column screws: (**A** Cut the skin about 3 cm, take the anterior inferior iliac spine as the needle entry point, and insert the needle in the direction of the sacroiliac joint; **B**–**D** obturator outlet position, obturator entrance position, and iliac oblique position determine that the guide needle does not penetrate the cortex; **E** Insert the screw along the guide pin)

The skin was incised longitudinally about 3 cm along the projection of the anterior inferior iliac spine and bluntly separated to the inferior iliac spine. Using this as a starting point, a guide needle is placed in the direction of the sacroiliac joint. The location of the guide pin in the iliac bone is determined by the closed-hole exit position, the closed-hole entrance position, and the oblique iliac position (Fig. 3). Note: 1. The incision can extend into the finger and touch the anterior inferior iliac spine, which is beneficial to determine the needle entry point; 2. The X-ray film at the closed-hole exit position clearly shows that the needle entry point is located in the center of the water drop, ensuring that the needle entry point is located between the inner and outer plates of the ilium.

#### Needle biopsy

**Fig. 4:**
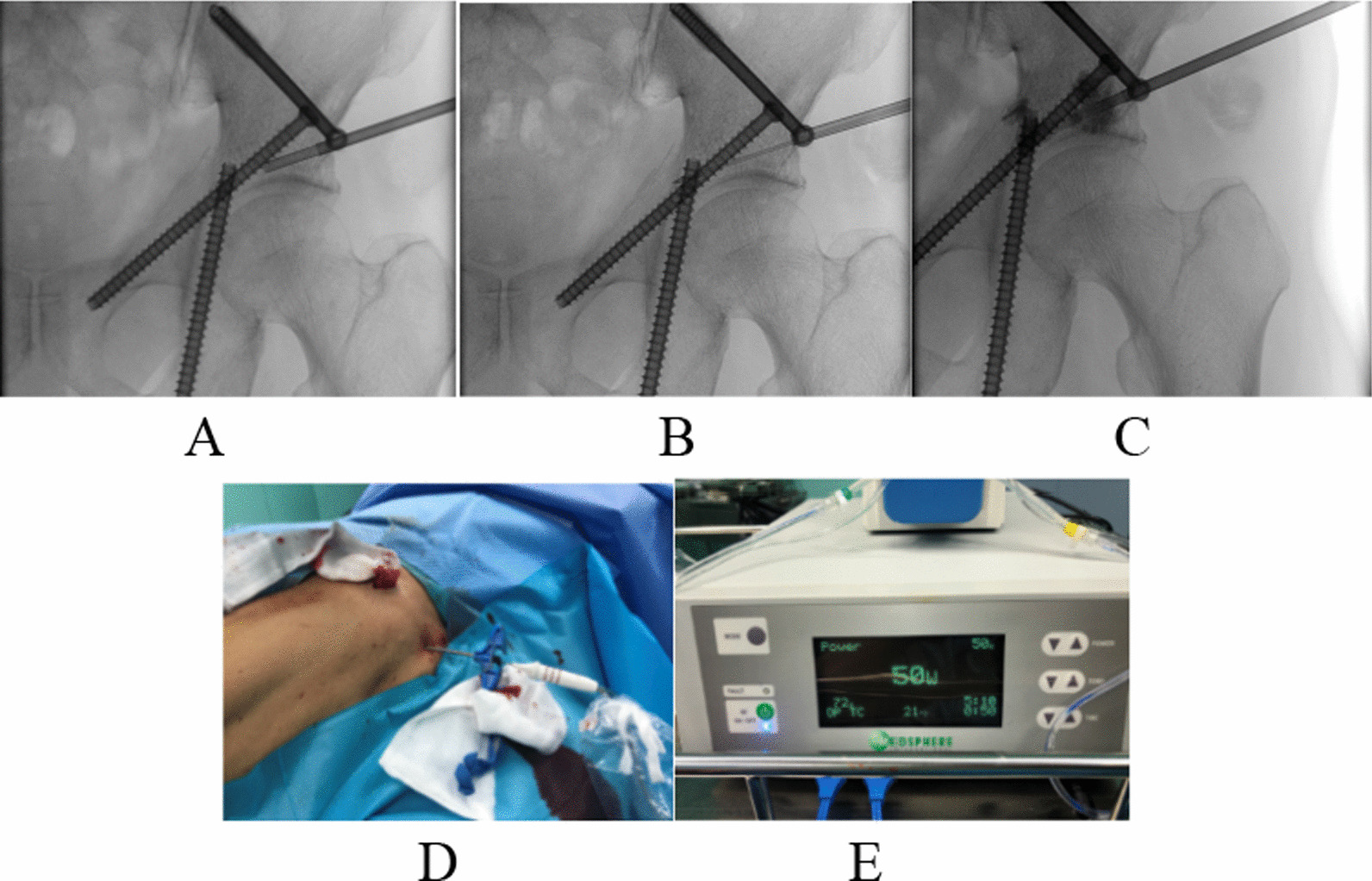
Puncture, radiofrequency, and bone cement injection all use the same channel (**A** needle biopsy; **B** Radiofrequency ablation; **C** bone cement injection; **D**, **E** Radiofrequency ablation intraoperative photo)

A puncture trocar needle is placed above the acanthus, and the C-arm machine is fluoroscopic to determine that the needle tip is located at the tumor site. The needle core is removed, the needle is inserted into the syringe for a needle biopsy, and the operation is repeated several times until a fine specimen is obtained (Fig. 4A).

#### Radiofrequency ablation

The electrode needle is inserted into the tumor through a secure channel established by the puncture trocar for radiofrequency ablation, and if the cancer is large, multiple multi-target ablations can be performed. The radiofrequency ablation temperature is set in the range of 80–100 °C, continuous ablation for 5 min, the radiofrequency ablation equipment adopts Shanghai Meide S-1500 radiofrequency ablation system, the instrument parameters are assigned to the frequency of 480 kHz, the power is 50W, and the electrode needle specification is 19G single needle (Fig. 4B, D, E).

#### Inject bone cement

Mix the bone cement, wait for the viscosity, slowly inject the lesion with a bone cement injector under the view of the C-arm machine, and slow down or stop the injection if leakage is found. The amount of bone cement injection is about 2–5 ml (Fig. 4C).

### Evaluation programs

Patients were observed pre- and postoperatively with Eastern Cooperative Oncology Group (ECOG) scores, visual analogue scale (VAS), Musculoskeletal Tumor Society (MSTS) scores, and function. A clinical osteopathic oncologist collected all data during the visit and the post-diagnosis follow-up.

### Statistical analysis

Data from patients completing the final follow-up were analyzed in this study. SPSS 23.0 software was used to analyze the data in the present study. Categorical variables are presented as frequencies and percentages, and continuous variables are presented as mean ± SD/mean ± SD (median). When continuous variables exhibit a normal distribution, a Student’s *t*-test was conducted to identify differences between the two groups. If not, the Mann–Whitney *U* test was employed. In the comparison, of categorical variables between groups, the Pearson chi-square test, and Fisher’s exact chi-square test were used. In all statistical analyses, statistical significance was accepted for *p* < 0.05.

## Results

A total of 11 patients with 13 hips were performed, of which two were bilateral, and the rest were unilateral. The average unilateral operation time was 167.4 min (148–193 min), and the average bleeding volume was about 20 ml (10–50 ml). The statistical and clinical features of the included patients are shown in Tables 1 and 2.

**Table 1:** Demographic characteristics of 11 patients

Cases	Age	Gender	Diagnosis	Preoperative treatment	Postoperative treatment		Fracture
					Bone protectant	Other treatments	
1	79	Male	Prostate cancer	NO	Zoledronic acid	NO	NO
2	53	Female	Bronchial or lung malignancy	NO	Zoledronic acid	Chemotherapy/Targeting	YES
3	72	Female	Bladder cancer	NO	Denosumab	Endocrinology/Targeting/Radiation Therapy	NO
4	64	Male	Prostate cancer	NO	Denosumab	Endocrine therapy	NO
5	62	Female	Lung cancer	NO	Zoledronic acid	Endocrine therapy	NO
6	53	Male	Lung cancer	NO	Zoledronic acid	Targeted therapy	NO
7	49	Female	Lung cancer	NO	Denosumab	Targeted therapy	NO
8	74	Male	Lung cancer	radiotherapy/chemotherapy	Denosumab	–	NO
9	58	Female	Breast cancer	endocrine therapy	Denosumab	Endocrine therapy	NO
10	73	Male	Prostate cancer	NO	Denosumab	NO	NO
11	50	Male	Nasopharyngeal cancer	NO	Denosumab	Chemotherapy	NO

**Table 2:** Comparison of preoperative and postoperative clinical evaluation indicators of 11 patients

Cases	operation time (minute)	Follow-up time (month)	MSTS	ECOG				VAS				Activity situation	
				Preoperative	Three days postoperative	Two months postoperatively	Last follow-up	Preoperative	Three days postoperative	Two months postoperatively	Last follow-up	Preoperative	Postoperative
1	193	7 (die)	13	3	2	3	3	8	5	5	6	Wheelchair	Bedridden
2	295 (bilateral)	24	23	4	3	2	2	9	4	2	2	Bedridden	Walking aids
3	170	8 (die)	22	4	3	2	2	8	5	5	3	Bedridden	Walking aids
4	185	22	26	3	2	1	1	7	5	2	2	Wheelchair	Walking alone
5	160	14	22	3	2	2	2	7	4	2	2	Bedridden	Walking aids
6	175	15	24	3	2	1	1	7	5	2	2	Wheelchair	Walking alone
7	310 (bilateral)	11	22	4	3	2	2	9	5	3	3	Bedridden	Walking aids
8	175	12	20	4	3	2	2	8	6	6	3	Bedridden	Walking aids
9	170	10	22	4	2	2	2	9	5	4	3	Bedridden	Walking aids
10	170	6	23	3	2	2	2	8	4	3	3	Wheelchair	Walking alone
11	173	3	21	3	2	1	1	7	3	2	2	Wheelchair	Walking alone

The follow-up of 11 patients was 3–24 months, with an average of 12 months, and the median follow-up time was 11 months. Two of the 11 cases died at the last follow-up, and 99 survived. Case 1 and Case 3 died 7 months and 8 months after surgery, respectively, for over 6 months. The survival time of the deceased patients was 7.5 months (range: 7–8 months), and the median survival time was 7.5 months.

**Fig. 5:**
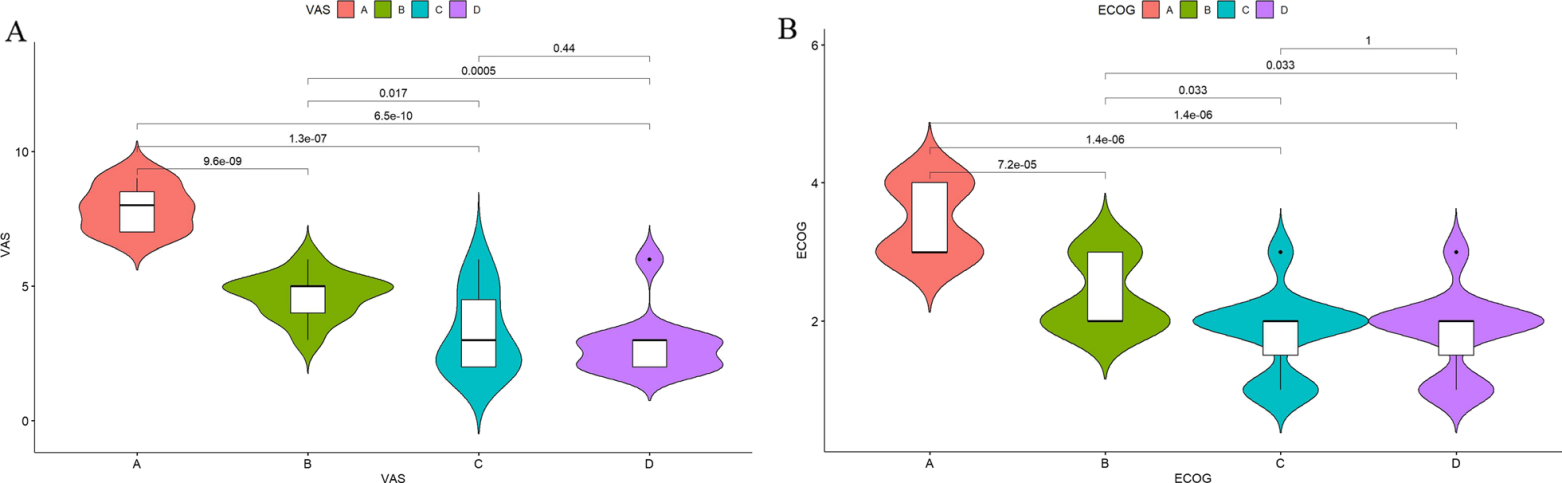
**A** Comparison of VAS scores before and after surgery. (A: preoperative; B: Three days after surgery; C: 2 months postoperatively; D: last follow-up) **B** Comparison of ECOG scores before and after surgery. (A: preoperative; B: Three days after surgery; C: 2 months postoperatively; D: last follow-up)

The mean preoperative VAS score of 11 patients was 7.91 (7–9), the mean postoperative VAS score was 4.64 (4–6) at three days, the mean postoperative VAS score was 3.27 (2–6) at 2 months, and the mean final VAS score was 2.82 (2–6). The pain was significantly reduced and showed continuous improvement compared with the preoperative pain (Fig. 5A).

Two patients had leakage of bone cement into the pelvis, and there were no apparent symptoms of discomfort at regular follow-ups after surgery. In one patient, the anterior column screw pierced the pubic end, causing perineal pain, and the pain improved after the surgical removal of the screw. No other complications, such as wound non-healing, infection, vascular nerve injury, and visceral injury, occurred in the remaining patients.

The average ECOG rating was 3.45 (grades 3–4) before surgery, 2.36 (grades 2–3) three days after surgery, 1.82 (grades 1–3) 2 months after surgery, and 1.82 (grades 1–3) at the last time (Fig. 5B).

The last MSTS93 functional score was 13–26 points, and the excellent rate was 81.82% (9/11). Case 1 did not undergo systemic antitumor therapy and local radiotherapy before and after surgery, and the lesions involved sacroiliac joints. The MSTS93 score was 13 points after surgery, and the average score of the remaining 10 cases was 22.5 points.

## Discussion

Patients with periacetabular metastases are often in the advanced stage of tumor treatment, facing the characteristics of advanced age, enormous tumor burden of the whole body, weak constitution, long anti-tumor treatment time, etc., poor general nutritional status, if the choice of tumor resection joint replacement, half pelvic replacement and other complex resection and reconstruction surgery, patients will face the following difficulties: (1) Long operation time, excessive intraoperative bleeding, some patients are difficult to tolerate surgical blows, and long postoperative recovery time; (2) It is easy to have postoperative complications such as wound non-healing, infection, and prosthetic dislocation; (3) The perioperative period often needs to stop systemic anti-tumor therapy, if complications such as wound infection occur after surgery, the systemic treatment time will be further delayed, which may lead to the progression of the primary tumor.

The interim follow-up results of Peking University People's Hospital in the clinical application of combined artificial hemipelvis prosthesis showed that the average intraoperative blood loss was 2762.6 ml. The postoperative complications were as high as 45%. The most common were poor wound healing (18%), deep infection rate (15%), and other complications, including fracture, prosthesis loosening, dislocation, etc. [7] Guo Wei et al. [8] retrospectively analyzed the clinical efficacy of 80 patients using a 3D printed artificial hemipelvis prosthesis and achieved the best results in the international use of artificial hemipelvis prosthesis reconstruction so far, the average MSTS score at the last follow-up was 83.9%. Still, the average blood loss of surgery was 1898.5 ml, the average operation time was 4.6 h, and there were 16 cases (20%) of postoperative complications, including 8 cases (10%) of wound non-healing. There were 5 cases of deep infection (6.3%) and 2 cases of dislocation (2.5%).

The main therapeutic goals of periacetabular metastases are pain reduction, rapid recovery, and improved quality of life [9]. Minimally invasive surgery is an option that can be performed without interrupting systemic antineoplastic therapy and minimizing potential complications such as wound nonunion and infection. The percutaneous “tripod” nail placement technique used in this study can be performed in an operating room with an X-ray machine, does not require a blood transfusion, and is safe and well tolerated by patients. The stable “tripod” structure around the acetabulum can significantly relieve patients’ postoperative pain, and they can ultimately bear weight or walk. The physiological load can promote local bone healing at the same time, reducing the occurrence of disuse osteoporosis and muscle atrophy. Our team’s early research using three-dimensional finite element technology found [10] that the local stress concentration of the acetabulum on the affected side was apparent. The stress-bearing effect after bone destruction was not good. The stress concentration of the acetabulum after percutaneous “tripod” surgery was relieved, which could achieve the supporting stress effect of normal bone. In this study, the preoperative VAS score of all 11 patients was severe pain, with an average ECOG rating of 3.45, a VAS score of 4.64, and an average ECOG score of 2.36 3 days after surgery. The pain was significantly improved, and they could stand with the assistance of a walker. The difference was statistically significant, the VAS score and ECOG rating were further enhanced in February after surgery (*p* < 0.05), and no rebound was seen at the last follow-up, which shows that percutaneous “tripod” surgery has a pronounced effect on pain relief and long-lasting effect.

Rui Yang et al. [6] combined local radiotherapy for postoperative patients with a “tripod.” Only one of the 11 patients in this study underwent radiotherapy after surgery, and the rest of the patients refused radiotherapy. Still, all patients were combined with bone protectants after surgery (denosumab 7 cases, zoledronic acid 4 cases). Through follow-up observation, case 1 did not undergo systemic treatment for the primary tumor, tumor progression, mild relief of postoperative pain, and death 7 months after surgery. The remaining ten patients were significantly relieved with postoperative pain, and cases 4 and 6 did not need to be treated with long-term pain medication at the 2-month postoperative follow-up. It can be seen that maintaining systemic anti-tumor therapy and giving bone protective measures after surgery can also achieve the purpose of pain relief even without radiotherapy.

Based on the principles of treatment of bone metastases [11], radiofrequency ablation was used in conjunction with cement filling. Since tripod surgery alone only acts as a stent to provide local temporary mechanical stability and bone metastases are not removed, the tumor will still grow further and destroy the surrounding bone. Therefore, we use local radiofrequency ablation to kill as many tumor cells as possible and reduce the tumor burden around the hip. Studies have shown [12, 13] that radiofrequency electrodes can coagulate and necrosis cancer cells through high temperature and can stimulate the body to produce cytokines such as tumor necrosis factor-α (TNF-α), interleukin-1 (IL-1), and interleukin-6 (IL-6) to inhibit the growth of cancer cells. After radiofrequency ablation, bone cement is injected into the upper acetabulum [14, 15], which is beneficial to mechanical conduction and support by enhancing the stiffness above the acetabulum, which effectively and immediately reduces the pain caused by local bone destruction[16, 17]; Secondly, the high temperature generated during bone cement polymerization also has a particular killing effect on cancer cells; Third, the injection of bone cement makes the screw fixation more secure. Therefore, the combination of radiofrequency ablation and bone cement filling during surgery can kill local tumor cells, reduce postoperative tumor progression, enhance immediate stability, and relieve pain, and radiofrequency ablation and bone cement filling share a channel, simple operation, short time-consuming, and our average intraoperative operation time are about 30 min. Of the eleven patients studied, postoperative imaging (both X-ray and CT) was conducted on seven. Among these, three exhibited tuberous bone formation within the acetabular lesion. Notably, none displayed significant progression of bone destruction (Additional file 1).

So far, only one patient (case 8) found that the distal end of the anterior column screw penetrated the cortex on the postoperative CT scan, causing perineal pain, and the penetrated screw was removed again by surgery. Due to the irregular curvature of the pubic branch, coupled with local osteolytic destruction, pathological fracture, and cortical bone discontinuity, it is easy to lose position when implanting guide needles. Therefore, we consider the front pillar pubic support screw to be the most challenging and recommend that this screw be placed first. We chose the upper extension of the tremendous trochanteric femur and the horizontal intersection of the anterior superior iliac spine towards the upper edge of the pubic symphysis. Still, due to individual bone differences and obesity, the guide needle could not be accurately positioned in the posterior upper acetabular bone cortex. Hence, before the guide needle enters the bone cortex, it is necessary to determine the position and direction of the needle entry point through multi-directional fluoroscopy and then further penetrate the guide needle. It is recommended to use full-threaded hollow nails to effectively thread fix with the remaining normal bone, enhancing stability and support. However, since the maximum length of full-threaded open nails we can obtain is only 100 mm when the nail size needs to exceed 100 mm, only half-threaded hollow screws can be used instead.

Because percutaneous guide needle positioning is easy to bend due to skin or muscle traction, which affects the needle direction, therefore, we use a puncture trocar needle for entrance positioning and establishing guide needle channel. The advantage is that the puncture trocar needle is hard in texture and is not easy to be bent by the surrounding muscle soft tissue; Secondly, after the puncture trocar needle is inserted into the bone cortex, the guide needle entrance and orientation are determined, which is not easy to loosen and change the direction, and its tube diameter (4.5 mm) can accommodate the penetration of the guide needle, and it is easy to determine the position of the guide needle combined with intraoperative fluoroscopy; Furthermore, it is easy to obtain tumor specimens through the puncture trocar channel, and it is easy to perform radiofrequency ablation and bone cement filling.

This study still has the following shortcomings: the study is a single-center retrospective study, the sample size is small, and it is difficult to combine the primary tumor type, systemic metastasis, bone protectant type, and other related factors for analysis; Second, there is a lack of controlled studies of other treatment modalities at the same time. However, compared with other previous studies on the treatment of periacetabular metastases, combined with the results of this study, it is enough to show that this procedure is a simple operation and significant efficacy treatment (Additional file 1).

## Conclusions

In summary, a percutaneous “tripod” combined with radiofrequency ablation and bone cement filling can enhance local stability while immediately relieving pain and long-term efficacy without affecting the systemic treatment of the patient’s primary tumor. This procedure can be used as a safe and effective minimally invasive treatment for patients with periacetabular metastases for patients who cannot tolerate open surgery.

### Supplementary Information

**Additional file 1**. Postoperative follow-up of patients with periacetabular metastases.

## Data Availability

The authors declare that the data supporting the findings of this study are available within the article.

## Abbreviations

ECOG: Eastern Cooperative Oncology Group
VAS: Visual analogue scale
MSTS: Musculoskeletal Tumor Society
