# Supplementary material for: Clinical efficacy analysis of percutaneous “tripod” combined with radiofrequency ablation and bone cement filling in the treatment of periacetabular metastases
Source: J Orthop Surg Res. 2023 Oct 11;18:767. doi: 10.1186/s13018-023-04255-w (PMC10565971; doi:10.1186/s13018-023-04255-w)
Supplement: Supplementary file 1 — Additional file 1. Postoperative follow-up of patients with periacetabular metastases. [file 13018_2023_4255_MOESM1_ESM.docx]

**Title:** Clinical efficacy analysis of percutaneous "tripod" combined with radiofrequency ablation and bone cement filling in the treatment of periacetabular metastases.

**Author information** : Yun Lan ^1,2△^, Ruoyu Li ^1△^ , Linheng Jiang ^2^, Nannan Zhou ^1^, Mincon He ^3^, Bin Fang ^1^, Chunzhi Yi ^1*^

1. Department of Orthopedic Oncology, the First Affiliated Hospital of Guangzhou University of Chinese Medicine, Guangzhou 510000, Guangdong, China
2. The First Clinical Medical College of Guangzhou University of Chinese Medicine, Guangzhou 510000, Guangdong, China
3. Guangdong Academy of Traditional Chinese Medicine Orthopedics and Traumatology, Guangzhou 510000, Guangdong, China

^△^Co-first author: Yun Lan, Ruoyu Li.

^*^Corresponding author：Chunzhi Yi , ORCID: 0000000329701575 E-mail: [531667142@qq.com](mailto:531667142@qq.com)

**Typical cases**


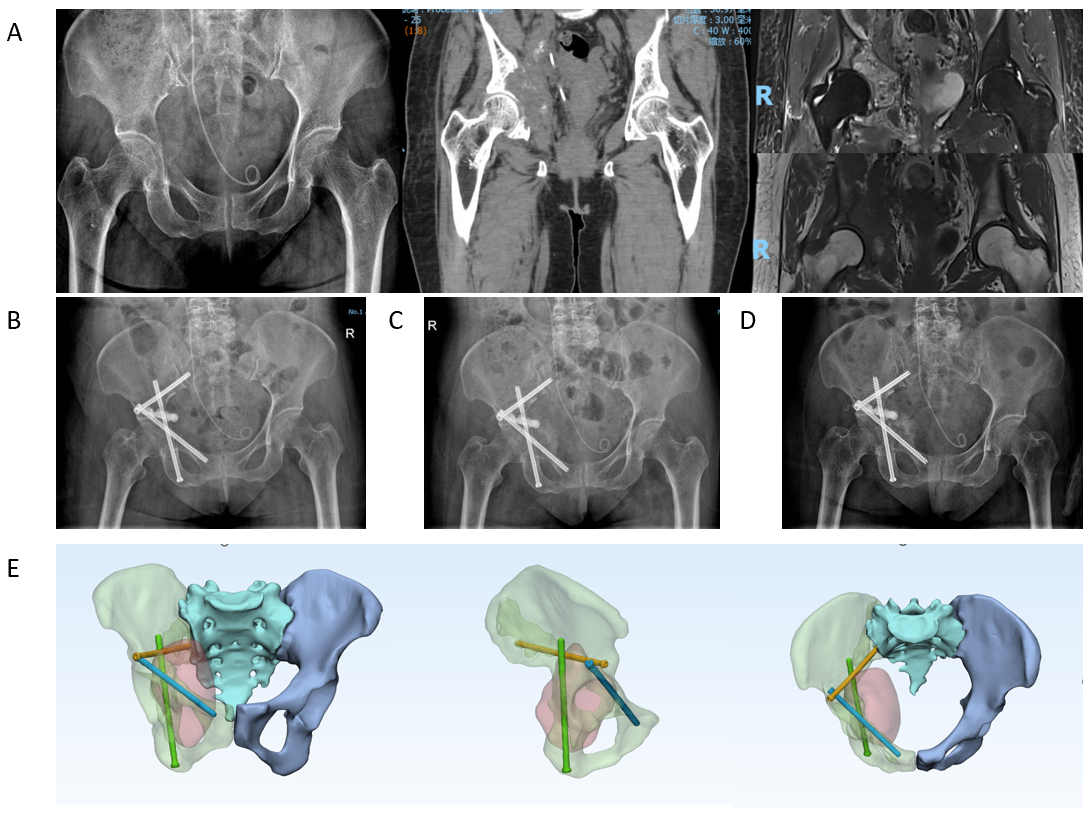


Figure 1 A 72-year-old female patient with a previous history of bladder cancer for more than 2 years and hip pain for 3 months was diagnosed with multiple metastases from bladder cancer. **A**: Preoperative lesion X-ray, CT and MRI, which showed osteolytic destruction of the inner wall of the patient's right acetabulum and above, with a soft tissue mass protruding into the pelvis; Harington type 3; **B**: X-rays 3 days after surgery showed good screw fixation and cement protruding into the pelvis along the lesion, and the patient had no obvious discomfort ;**C**: Postoperative 2-month x-ray, no change in screw position and osteogenic changes within the medial acetabular lesion; **D**: On postoperative X-ray review in 6 months, the internal fixation was secure and the local lesion was further osteogenic; **E**: Post-operative 3D reconstruction schematic, which can understand the position of the screw from different angles.


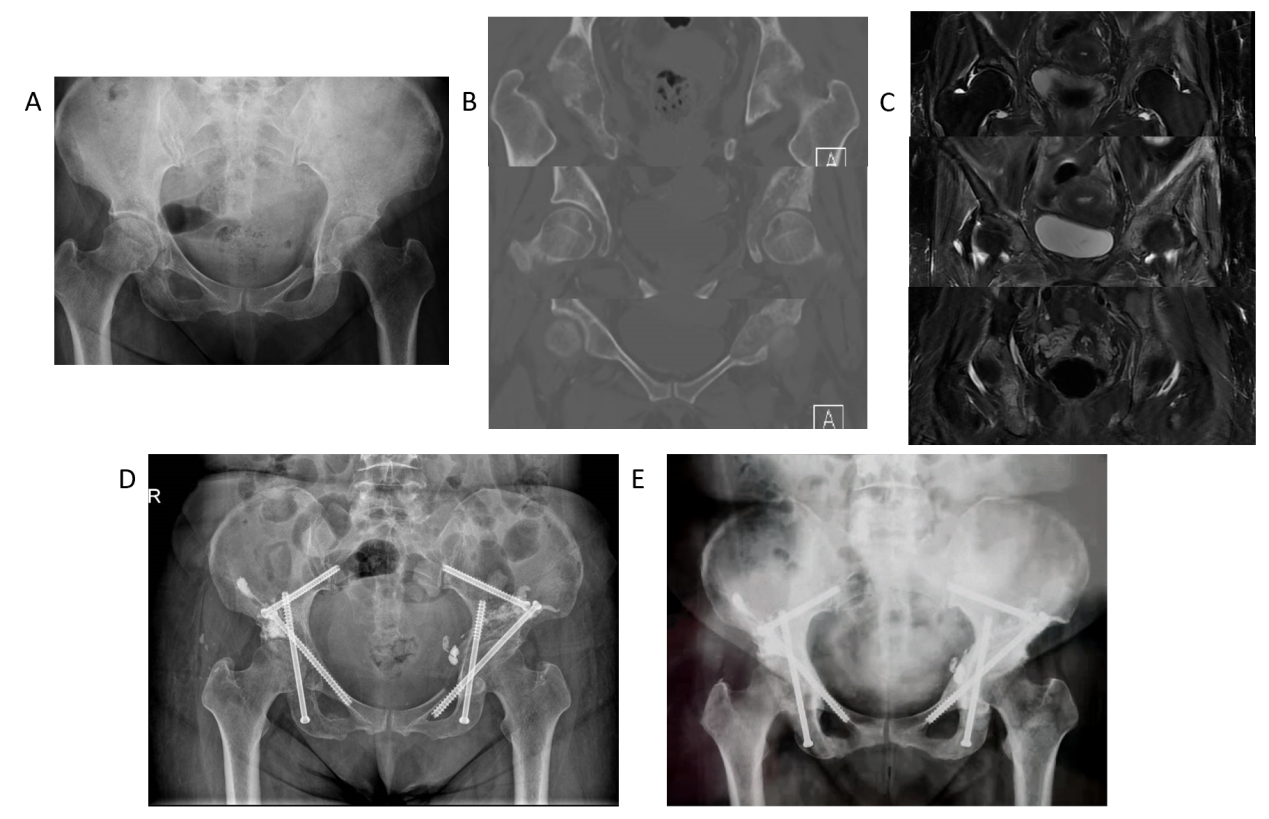


Figure 2 A 54-year-old female patient with a history of lung cancer for 1 year, bilateral hip pain and limited mobility for 4 months, was diagnosed with multiple metastasis of lung cancer. A, B, C: X-ray, CT, MRI at the affected area before surgery, bilateral osteolytic destruction of the medial wall and upper acetabulum, all Harington type 3; D: X-ray 1 day after surgery, bilateral acetabulum, iliac screw fixation and bone cement filling, a slight bone cement exudation from the inner edge of the left acetabulum, and the position of both hip joints is normal; E: X-rays were re-examined three months after surgery, and the screw position did not change, and the osteogenesis of the lesion changed.


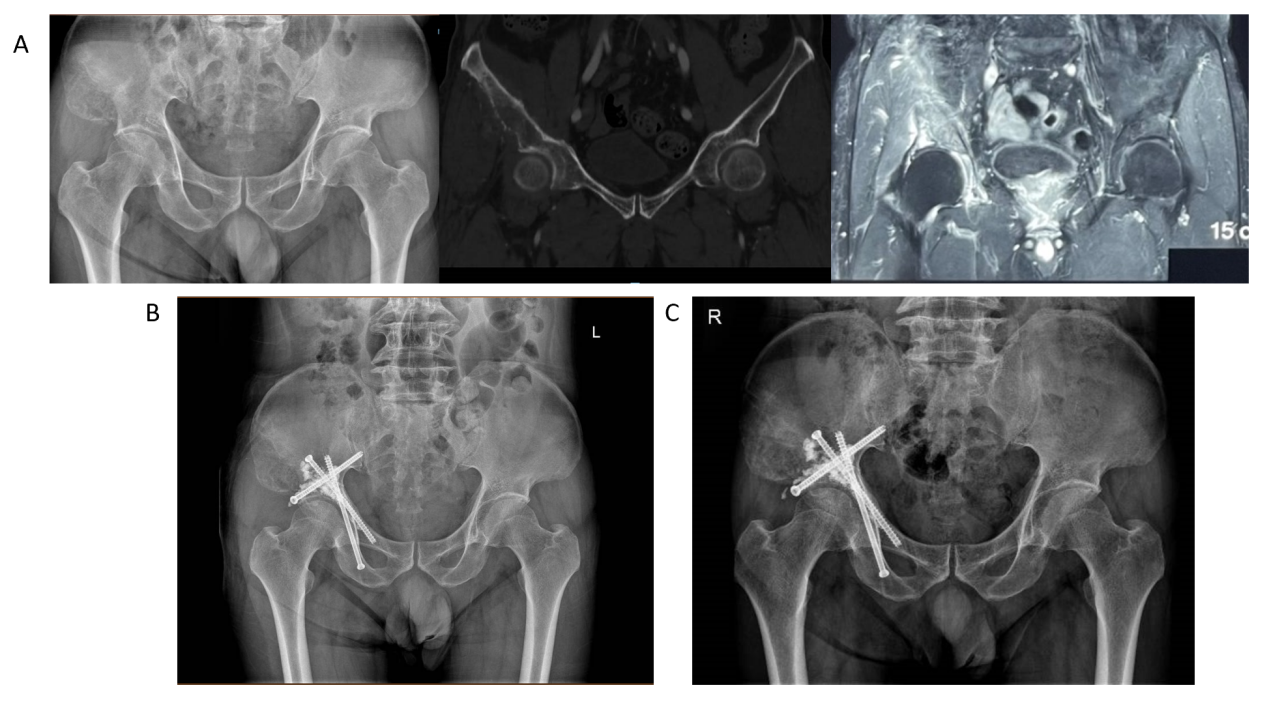


Figure 3 A 64-year-old male patient with a history of prostate cancer for more than 1 year, right hip pain and limited mobility for 1 year, was diagnosed with prostate cancer and metastases to the right pelvis.A: X-ray, CT, and MRI of the affected area before surgery, visible osteolytic destruction above the right acetabulum and inner wall, Harington type 3; B: X-ray 3 days after surgery, it can be seen that the screw position is good, and the bone cement is evenly diffused above the acetabulum; C: X-ray was re-examined two months after surgery, the screw position did not change, and the local osteogenesis of the lesion above the acetabulum changed.
